# Impact of Channel Thickness and Doping Concentration for Normally-Off Operation in Sn-Doped *β*-Ga_2_O_3_ Phototransistors

**DOI:** 10.3390/s24175822

**Published:** 2024-09-07

**Authors:** Youngbin Yoon, Yongki Kim, Myunghun Shin

**Affiliations:** School of Electronics and Information Engineering, Korea Aerospace University, Goyang 10540, Republic of Korea; ybyoon93@gmail.com (Y.Y.); ygk1740@gmail.com (Y.K.)

**Keywords:** gallium oxide (Ga_2_O_3_), enhancement-mode MOSFET, phototransistor, spin-on-glass (SOG) doping, computer-aided design (TCAD) simulations

## Abstract

We demonstrate a Sn-doped monoclinic gallium oxide (*β*-Ga_2_O_3_)-based deep ultraviolet (DUV) phototransistor with high area coverage and manufacturing efficiency. The threshold voltage (*V*_T_) switches between negative and positive depending on the *β*-Ga_2_O_3_ channel thickness and doping concentration. Channel depletion and Ga diffusion during manufacturing significantly influence device characteristics, as validated through computer-aided design (TCAD) simulations, which agree with the experimental results. We achieved enhancement-mode (e-mode) operation in <10 nm-thick channels, enabling a zero *V*_G_ to achieve a low dark current (1.84 pA) in a fully depleted equilibrium. Quantum confinement in thin *β*-Ga_2_O_3_ layers enhances UV detection (down to 210 nm) by widening the band gap. Compared with bulk materials, dimensionally constrained optical absorption reduces electron–phonon interactions and phonon scattering, leading to faster optical responses. Decreasing *β*-Ga_2_O_3_ channel thickness reduces *V*_T_ and *V*_G_, enhancing power efficiency, dark current, and the photo-to-dark current ratio under dark and illuminated conditions. These results can guide the fabrication of tailored Ga_2_O_3_-based DUV phototransistors.

## 1. Introduction

Gallium oxide (Ga_2_O_3_) has received significant attention in the field of power semiconductors owing to its wide energy bandgap (4.9 eV) and potential for producing single-crystal wafers via the melt growth method [1]. The wide band gap of Ga_2_O_3_ facilitates the absorption of deep ultraviolet (DUV) wavelengths below 280 nm, making it a prominent material for DUV detector devices. Its exceptional photoelectric conversion efficiency, owing to its nearly direct energy-band structure, further enhances its appeal as an optical device [2,3,4]. DUV radiation is invisible to the human eye and often associated with harmful signals, necessitating constant monitoring. Applications such as fire detection, corona detection, transmission tower failure detection, rocket combustion monitoring, and aircraft composite material curing rely on long-term continuous monitoring, for which the power consumption of the device is crucial [5,6,7]. A low dark current is advantageous for this purpose. Additionally, manufacturing large-area sensors is vital for covering various light source angles, providing accurate and reliable data, and increasing the efficiency and versatility of the sensors [8,9]. Capturing more light enhances sensor sensitivity, facilitates the detection of low-intensity light or subtle changes, and improves the signal-to-noise ratio. Because most ultraviolet sources on Earth are artificial and harmful to humans, a wide-area detection capability is critical for protection in hazardous environments. Applications include environmental monitoring, security systems, and medical diagnostics.

Recent advancements in Ga_2_O_3_-based DUV sensors have led to research on various structures, such as metal–semiconductor–metal (MSM) photodetectors [10,11,12]; *p*-type, intrinsic, and *n*-type (PIN) photodiodes (PD) [13,14,15]; Schottky barrier diodes (SBDs) [16,17,18]; heterojunction PDs [19,20,21]; and phototransistors [22,23,24,25]. Ga_2_O_3_ photodetectors are utilized in single-crystal, polycrystalline, and amorphous forms, with successful reports of sensors featuring light-receiving elements with nanomaterials such as nanorods and fibers [26,27,28]. While it is easier to manufacture two-terminal devices like PDs because of their simple structures, they face issues such as high dark currents and limited control over currents and photosensitivity. 

Three-terminal phototransistors offer several advantages in this regard. Gate control allows for light current modulation, enhanced light sensitivity through the inherent gain of the field-effect transistor (FET), and the ability to turn off the FET channel, resulting in a low dark current and a high signal-to-noise ratio. Consequently, FET-based phototransistors are advantageous in terms of power consumption when operated at equilibrium, necessitating enhancement-mode (e-mode) operation with normally-off characteristics. 

Achieving an e-mode operating transistor typically involves the depletion of a p–n structure [29]. Unfortunately, the fabrication of *p*-type Ga_2_O_3_ is challenging [30,31,32]. Most metal-oxide semiconductors, including Ga_2_O_3_, In_2_O_3_, SnO_2_, and TiO_2_, exhibit *n*-type doping because of the increased electron concentration from the oxygen vacancies formed during material growth [33,34]. Additionally, owing to the 2p orbital of oxygen and the solid ionic bonds between the metal and oxygen atoms, the small valence band curvature of metal oxide semiconductors results in localized electron clouds and limited band dispersion [35]. This makes it difficult to achieve usable levels of *p*-type conductivity despite various efforts to create *p*-type metal oxide semiconductors. Because of these challenges, active research has focused on developing Ga_2_O_3_ FETs capable of e-mode operation without relying on p–n depletion structures. Proposals such as n-doped [36] and gate-recess [37,38,39] type devices have shown promise but require cost-effective solutions. Controlling the channel thickness of Ga_2_O_3_ FETs can change the threshold voltage (*V*_T_) [40,41] and carrier concentrations in the channel of TFTs and make the TFTs operate in the normally-off mode (enhancement mode) [41]; however, the impact of channel thickness and doping concentration on normally-off operation is not yet fully understood and requires investigation.

In this study, we propose a large-area Ga_2_O_3_-based phototransistor that is both power- and process-efficient. We optimized a polycrystalline *β*-Ga_2_O_3_ phototransistor from depletion-mode (d-mode) to e-mode, with threshold voltage (*V*_T_) modulation achieved through Sn doping and channel thickness adjustments. We conducted a technology computer-aided design (TCAD) simulation to analyze the Ga diffusion effect and to understand e-mode operation for a thin-channel *β*-Ga_2_O_3_ phototransistor. We characterized the electrical and optical performance of the *β*-Ga_2_O_3_ phototransistor and demonstrated its potential application for high-performance DUV detectors. Section 2 presents the fabrication and characterization method of Ga_2_O_3_-based phototransistor. Section 3 provides detailed results and discussions on the physical and electrical properties of the Ga_2_O_3_ channel, TCAD simulation results, and photoresponse characteristics.

## 2. Materials and Methods

Amorphous Ga_2_O_3_ thin films were deposited using a Ga_2_O_3_ target via radio frequency (RF) sputtering at 70 W in an Ar atmosphere at a pressure of 1.3 mTorr. These films were then crystallized into monoclinic *β*-Ga_2_O_3_ by annealing at 900 °C for 1 h in an atmospheric environment. To create *n*-type *β*-Ga_2_O_3_ thin films, Sn atoms were introduced using the spin-on-glass (SOG) technique. For SOG doping, a solution of SnCl_4_ (99.995%, 2.226 g/mL) and 2-methoxyethanol (2MOE) was prepared and stirred at 60 °C for 1 h. The doping concentration was controlled by varying the amount of SnCl_4_. The spin coating process involved spinning the solution at 500 rpm for 60 s, followed by 3000 rpm for 6 s. The coated films were then heated at 120 °C for 120 s and subsequently at 300 °C for 600 s in air to remove the solvent. Finally, the polycrystalline *β*-Ga_2_O_3_ thin films were annealed at 450 °C for 1 h in an N_2_ atmosphere to drive in the dopants. Source/drain electrodes comprising Ti/TiN (5/150 nm) were fabricated on the polycrystalline *β*-Ga_2_O_3_ thin films using standard lithography and a subsequent lift-off process. The substrates, composed of p^++^-Si and SiO_2_, were used as the back-gate electrode and gate dielectric, respectively. 

The thickness and crystal structure of the Sn-doped polycrystalline *β*-Ga_2_O_3_ phototransistors were analyzed using TEM (EM-ARM200F, JEOL, Ltd., Tokyo, Japan) at an accelerating voltage of 300 keV. The cross-sectional samples were prepared using a focused ion beam milling system (Helios 5 UX, Thermo Fisher, Waltham, MA, USA). FFT patterns for crystal structure analysis were obtained with a Gatan DigitalMicrograph^®^ (AMETEK, Inc., Berwyn, PA, USA). The transmittance of the *β*-Ga_2_O_3_ layers was measured using an ultraviolet-visible–near-infrared spectrophotometer (UV-3600, Shimadzu, Kyoto, Japan). The dark currents and photocurrents of the Sn-doped polycrystalline *β*-Ga_2_O_3_ phototransistors were recorded with a semiconductor parameter analyzer (Keithley 4200-SCS, Tektronix, Beaverton, OR, USA). Optical stimulation was provided using a monochromatic light source comprising a lamp (6269, Newport, Irvine, CA, USA), a lamp housing (66921, Newport, RI, USA), a monochromator (CS130B-1-MC, Newport, RI, USA), and an arc lamp power supply (69920, Newport, RI, USA). Monochromatic optical pulses were controlled using transistor–transistor logic (TTL) inputs via a function generator (AFG-2225, GW Instek, New Taipei City, Taiwan) connected to diaphragm shutters (SHB025T, Thorlabs, Newton, NJ, USA). 

## 3. Results and Discussion

**Figure 1:**
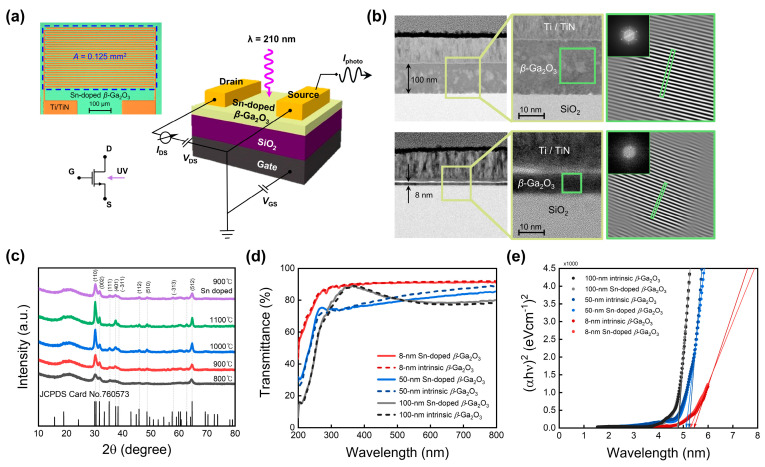
(**a**) Schematic and optical micrograph and (**b**) cross-sectional transmission electron microscopy (TEM) images and their fast Fourier transform (FFT) patterns for the Sn-doped polycrystalline *β*-Ga_2_O_3_ deep ultraviolet (DUV) phototransistor. (**c**) X-ray diffraction (XRD) analysis results according to crystallization annealing temperature and Sn doping of Ga_2_O_3_ with a thickness of 100 nm. (**d**) Transmittance and (**e**) Tauc plot according to the presence or absence of *β*-Ga_2_O_3_ thin film doping with thicknesses of 100, 50, and 8 nm.

The schematic in Figure 1a shows that the Sn-doped polycrystalline *β*-Ga_2_O_3_ DUV phototransistor was fabricated in a back-gated stack structure. The source and drain electrodes were fabricated using Ti/TiN, the gate insulator was SiO_2_, and the gate was a *p*^++^-Si substrate. It has a typical phototransistor structure in which the photocurrent is output through the drain terminal when light is irradiated onto the top of the device. The upper left corner of Figure 1a shows an optical micrograph of the fabricated device. To maximize the width of the transistor within the same area, a pattern was designed in which the source and drain electrodes were interlocked in the form of pods. The length and width of the fingers are 500 and 5 μm, respectively, and the number of fingers on the electrode pad on one side is 13. The gap between the two crossed fingers to which the field is applied is designed to be 5 μm. The light-receiving area of the device, which is defined by the rectangular area indicated by the dotted line, was 0.125 mm^2^. Transmission electron microscopy (TEM) analysis was used to investigate the structural stability and crystalline properties of the fabricated device. Figure 1b shows the cross-sectional TEM images and fast Fourier transform (FFT) patterns of devices with Ga_2_O_3_ channel thicknesses of 100 and 8 nm, respectively. The FFT pattern of a square section randomly selected from the Ga_2_O_3_ region of the cross-sectional image was obtained, and the lattice spacing was extracted. The obtained lattice spacing values were selected and presented in Table 1, matching the monoclinic Ga_2_O_3_ JCPDS (reference code: 76-0573) within an error range of ±2%. Consistently, various planes were observed at thicknesses of 100 and 8 nm. Consequently, Ga_2_O_3_ exhibits the *β*-phase in the device and exists in a polycrystalline form. 

Figure 1c shows the X-ray diffraction (XRD) analysis results for Ga_2_O_3_ subjected to various crystallization heat-treatment temperatures and Sn doping. Ga_2_O_3_ thin films deposited via sputtering have the advantage of large-area deposition; however, crystallization annealing is required to obtain device stability because they become amorphous immediately after deposition. *β*-Ga_2_O_3_ is a thermally and chemically stable phase, requiring high-temperature annealing to form the *β*-phase. Annealing was performed for 1 h in an Ar atmosphere with temperatures ranging from 800 to 1100 °C (black, red, blue, and green curves). As shown in the XRD results, increasing the annealing temperature improved the crystallinity and increased the peak intensity. Considering process efficiency (under 1000 °C), 900 °C was selected as the optimal crystallization annealing temperature. After crystallization annealing, a SOG process was used for Sn doping. Figure 1c presents the results for an SOG doping concentration of 0.025 M. Detailed information is provided in the experimental section. The purple curve represents the case where SOG doping was applied after crystallization heat treatment at 900 °C. The XRD peak results show that the *β*-phase was normally formed even after doping. Figure 1d,e show the transmittance and Tauc plot, respectively, for *β*-Ga_2_O_3_ thin films with thicknesses of 100, 50, and 8 nm, with and without doping. The results are consistent with the typical optical properties of *β*-Ga_2_O_3_, which exhibits near-transparency in the visible light region and absorbs light in the ultraviolet region. 

The optical bandgap of each sample was estimated by extrapolating from the take-off area of the Tauc plot. For the intrinsic films with thicknesses of 100, 50, and 8 nm, the estimated band gaps were 4.82, 5.25, and 5.45 eV, respectively. The bandgaps of the Sn-doped films were 4.79, 5.13, and 5.31 eV, respectively. A consistent bandgap reduction was observed in the doped samples. This was due to the creation of a doping level at the bottom of the conduction band by doping with Sn, which acted as a donor. Additionally, as the thickness of Ga_2_O_3_ decreased, the band gap increased, which can be interpreted as a quantum confinement effect as the thin film thickness decreased. Consequently, it was confirmed that the phototransistor composed of Sn-doped *β*-Ga_2_O_3_ channels is suitable for absorption in the DUV region. 

**Figure 2:**
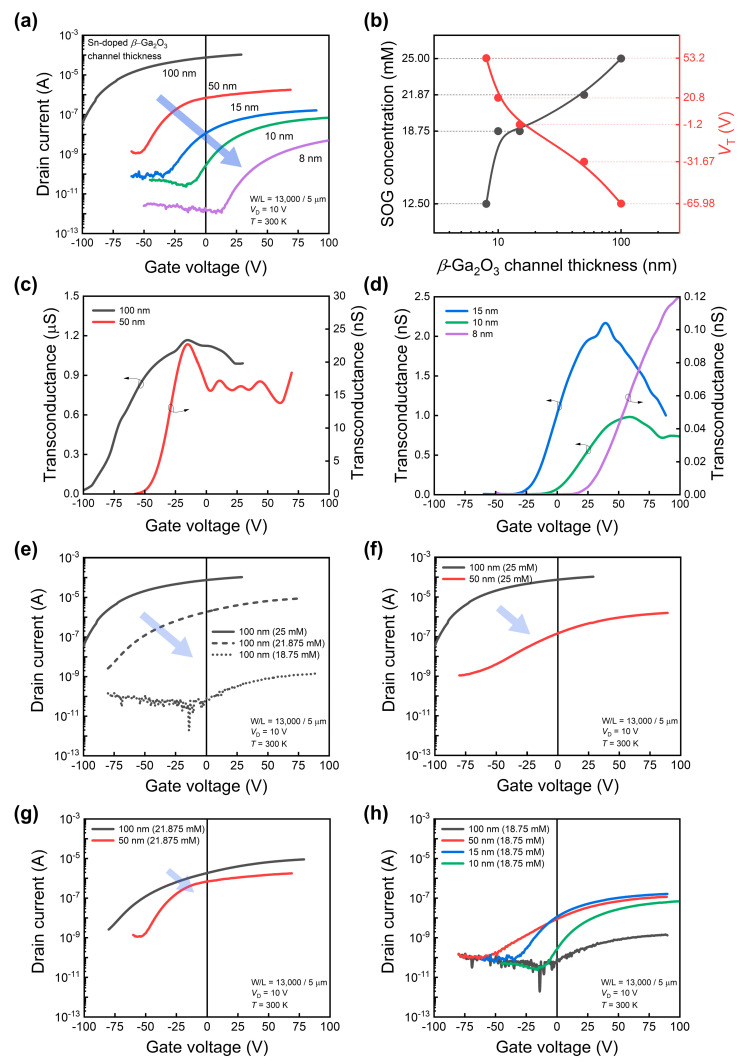
(**a**) Transfer curves of Sn-doped *β*-Ga_2_O_3_ phototransistors fabricated with *β*-Ga_2_O_3_ channel thicknesses of 100, 50, 15, 10, and 8 nm. (**b**) Optimal spin-on-glass (SOG) doping concentration and corresponding V_T_ change according to *β*-Ga_2_O_3_ channel thickness. Transconductance for each thickness (**c**) 100 and 50 nm, (**d**) 15, 10 and 8 nm. Each data point was calculated from the curves in (**a**). (**e**) Changes in transfer curves with a constant thickness of 100 nm and varying SOG doping concentrations. Changes in transfer curves for a constant SOG doping concentration and varying thickness at 100 nm and 50 nm (**f**) 25 mM, (**g**) 21.875 mM. (**h**) Transfer curves for a constant SOG doping concentration at 18.75 mM and varying thickness at 100, 50, 15, and 10 nm.

Next, we investigated the electrical characteristics of the fabricated Sn-doped *β*-Ga_2_O_3_ phototransistor under dark conditions. Figure 2a shows the transfer curves in the dark for Sn-doped *β*-Ga_2_O_3_ phototransistors fabricated with *β*-Ga_2_O_3_ channel thicknesses of 100, 50, 15, 10, and 8 nm, respectively. As the *β*-Ga_2_O_3_ channel thickness decreases, the transfer curve shifts downward, and the *V*_T_ of the phototransistor changes from a negative to a positive value. Here, *V*_T_ is extracted as the x-axis (gate voltage) intercept value of the extrapolated line drawn at the maximum drain current slope point on the transfer curves on a linear scale. In other words, the device transitions from d-mode to e-mode operation. This can be interpreted as an effect of the channel thickness, assuming that the doping concentration range of the devices at each thickness is similar. When different materials are stacked in a device, the energy-band structure is aligned based on the Fermi level at equilibrium. Owing to the difference in work functions between the bottom p^++^-Si gate and *β*-Ga_2_O_3_, depletion occurs at the interface where the *β*-Ga_2_O_3_ channel, which has a relatively small carrier concentration, contacts SiO_2_. This depletion thickness remains constant near the interface even if the channel thickness changes, assuming that the doping concentrations are similar. If the channel is sufficiently thick (e.g., 100 nm), neutral and *n*-type regions appear at a distance from the interface boundary. However, as the channel thickness decreases, the depletion region dominates the entire channel, gradually causing *V*_T_ to shift in the positive direction. Consequently, the 8 nm-thick channel remains fully depleted at equilibrium, achieving e-mode operation with a positive *V*_T_. 

The SOG doping process involves spin-coating an SOG solution containing a Sn source on the surface, followed by drive-in annealing. Devices with different *β*-Ga_2_O_3_ thicknesses require optimization of the SOG solution concentration suitable for each thickness condition. The results of the optimization of the molar concentration of the SOG doping solution for various thicknesses are presented in Table 2. The higher the molar concentration, the higher the amount of the Sn source. The optimal concentration of the SOG solution for a 100 nm thickness was 25 mM, while the optimized concentrations for 50, 15, 10, and 8 nm were 21.875, 18.75, 18.75, and 12.5 mM, respectively. By converting these molar values into weights, the values were found to be 0.875, 0.75, 0.75, and 0.5 based on a thickness of 100 nm. Figure 2b shows the optimal SOG concentration and *V*_T_ change according to the *β*-Ga_2_O_3_ channel thickness. It was confirmed that the optimal SOG concentration had a non-linear correlation with the *β*-Ga_2_O_3_ channel thickness. Transfer curves were compared for a more detailed analysis by maintaining either the *β*-Ga_2_O_3_ thickness or the SOG doping concentration and varying the other parameters. Figure 2c,d show the transconductance for each thickness extracted from the transfer curves in Figure 2a.

Figure 2e represents the case with the *β*-Ga_2_O_3_ thickness constant at 100 nm and a varying SOG doping concentration. As the doping concentration decreases, the curves shift downward to the right, and *V*_T_ becomes positive. This is likely due to an increase in the depletion area of the channel as the doping concentration decreases. In addition, the slope of the transfer curves corresponding to the current take-off gradually decreases. As the carrier concentration in the channel decreased, the channel and contact resistances increased. Figure 2f–h show the results for a constant SOG doping concentration and varying *β*-Ga_2_O_3_ thickness. In Figure 2f,g, the transfer curves shift downward to the right as the thickness decreases. Figure 2h illustrates the cases for an SOG doping concentration of 18.75 mM and a *β*-Ga_2_O_3_ thickness of 100, 50, 15, 10, and 8 nm. The 100 nm device has the lowest current owing to insufficient doping relative to its thickness. The 50 nm device also shows a high-resistance channel with a relatively flat slope. The 15 and 10 nm curves are relatively steep, indicating an appropriate doping concentration level. In this case, the transfer curves tend to shift downward to the right as the thickness decreases from 15 to 10 nm. It was confirmed that *V*_T_ varied depending on the channel thickness and doping concentration. Additionally, because the channel resistance changes with the doping concentration, to obtain a positive *V*_T_ without a loss of channel current, an additional process, such as applying local doping in the source–drain region, must be implemented to achieve e-mode operation.

**Figure 3:**
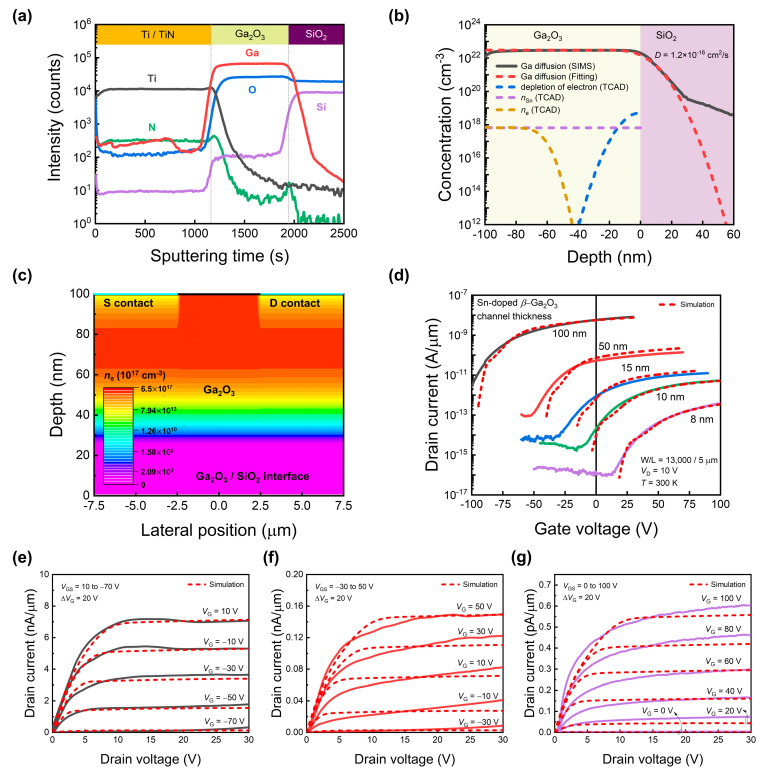
(**a**) Secondary ion mass spectrometry (SIMS) analysis results of 100-nm thick *β*-Ga_2_O_3_ device subjected to crystallization annealing at 900 °C. (**b**) Ga diffusion profile fitted with a complementary error function (ERFC) and depletion of electrons caused by Ga diffusion, electron concentration by SOG doping (*n*_Sn_), final electron concentration profile (*n*_e_) implemented through technology computer-aided design (TCAD) device simulation. (**c**) Color contours of the *n*_e_ of 100 nm-thick Sn-doped *β*-Ga_2_O_3_ channel. Result of matching for each *β*-Ga_2_O_3_ thickness in a dark room through TCAD device simulation (**d**) transfer and (**e**–**g**) output curves.

Next, the results of analyzing the transfer curves of Sn-doped polycrystalline *β*-Ga_2_O_3_ phototransistors in the dark, using TCAD device simulation, are presented. To model the device’s *V*_T_ change depending on the Ga_2_O_3_ channel thickness, the measured value could not be matched simply by channel depletion. Therefore, we considered the impact of Ga diffusion during the device fabrication process. Figure 3a shows the secondary ion mass spectrometry (SIMS) depth profile in the layered structure after a 900 °C crystallization annealing process. From the interface, Ga diffuses into SiO_2_. The lack of Ga in Ga_2_O_3_ can be interpreted as reducing the electron concentration. This effect may reduce the electron concentration in the Ga_2_O_3_ channel region adjacent to the SiO_2_ interface and affect the threshold voltage. For bulk materials with sufficiently thick channels, the influence of these interfaces is minimal. However, thinner materials have a larger surface-to-volume ratio and are significantly affected by surface and interface phenomena. Based on this analysis, we prepared the doping profile (TCAD profile in Figure 3b) and performed the TCAD simulation to understand the device operation. The concentration of Ga diffused into the SiO_2_ decreases steadily from the interface and exhibits a sudden change in gradient at an intermediate point. The region with a steep gradient before the inflection point is generally interpreted as direct volume diffusion, while the region with a shallow gradient after the inflection point is interpreted as grain boundary diffusion [42,43]. Since the direct volume diffusion region shows a significant concentration level, we focused on fitting the diffusion equation to this region. The complementary error function (ERFC) diffusion equation was applied to reflect the diffusion of Ga in the simulations. Equation (1) represents the concentration profile (*N*(*x*, *t*), atoms/cm^3^) used in Ga diffusion modeling.

(1) $$N\left(x,\;t\right)=N_{0}erfc[-\frac{x}{2\sqrt{Dt}}]$$

where *x* is depth position (nm), *D* is the temperature-dependent diffusivity (cm^2^/s), and *t* is the diffusion time (s). Figure 3b shows the result of fitting the Ga diffusion profile obtained from SIMS to the ERFC diffusion equation. From the previous TEM analysis, we determined the thickness of each stacked material, allowing us to approximate the sputtering time in nanometers. However, since the sputtering rates differ for each material, reference tests for each material with known thicknesses are necessary to obtain an accurate depth. The results of fitting the diffusion equation are shown as red dashed lines. The diffusivity corresponding to this curve was found to be 1.2 × 10^−16^ cm^2^/s. The dashed lines illustrate the dopant and electron concentrations due to Ga diffusion, as reflected in the TCAD simulation.

To account for the effects of Ga diffusion into SiO_2_ and the depletion of electrons in the Ga_2_O_3_ channel, we assumed a *p*-type counter-doping scenario from the Ga_2_O_3_/SiO_2_ interface into the Ga_2_O_3_ region. This results in electron depletion of the Ga_2_O_3_ channel in the thin sections adjacent to the interface. As Ga diffuses into SiO_2_, Ga deficiency occurs in the Ga_2_O_3_ channel, which is indicated by the blue dashed depletion of the electron curve. *n*_Sn_ represents the electron concentration in the channel caused by SOG doping. Previous research has confirmed that the Sn concentration in the channel remains constant during SOG doping; accordingly, *n*_Sn_ is assumed to have a continuous value in the channel [44]. The concentration at the starting point where electron depletion occurs was optimized to a value between the maximum concentration of Ga and *n*_Sn_. The final electron concentration (*n*_e_) profile, reflecting the combined effects of SOG doping and Ga diffusion, is shown as a dotted ocher line. These analyses were applied to TCAD simulations, and the materials and physical parameters used are presented in Table 3.

The Shockley–Read–Hall (SRH) model, which is fundamental for metal–oxide–semiconductor FET simulation, was used, and the energy band gap values were derived from previous transmittance measurements. Figure 3c shows the simulated color contours of the *n*_e_ of a 100 nm-thick Sn-doped *β*-Ga_2_O_3_ channel. The electron concentration decreases exponentially as a function of distance from the channel surface, and the Ga_2_O_3_/SiO_2_ interfacial channel becomes completely depleted at approximately 20 nm. Figure 3d shows the results of matching the transfer curves for each thickness of *β*-Ga_2_O_3_ in a dark room through the TCAD device simulation, which matches well with the measured results. The donor concentration, which most directly affects *V*_T_, was optimized to 6.5 × 10^17^ cm^−3^ for the 100 nm device and appropriately scaled for the 50, 15, 10, and 8 nm devices by adjusting the SOG doping concentration weight ratio. The theoretical limit of electron mobility in single-crystal *β*-Ga_2_O_3_ is 300 cm^2^/Vs [49]. However, in this study, the Ga_2_O_3_ channel formed is a polycrystalline thin film. Thus, lattice scattering is accompanied by scattering due to grain boundaries and defects. Additionally, since Ga_2_O_3_ has extremely low thermal conductivity (10~30 W/m K) [45], mobility degradation can also occur due to internal heat accumulation [50,51]. Considering these causes of mobility degradation, the value was optimized to be lower than the ideal value. The fabricated Sn-doped *β*-Ga_2_O_3_ phototransistors exhibited somewhat leaky SiO_2_ due to the high-temperature crystallization annealing process at 900 °C. Consequently, the off-current of the device is similar to the gate leakage current. Although this leakage current was not reflected in this simulation, it can be optimized by applying a gate leakage current model. Figure 3e–g show the measured output characteristic curves and simulation matching results for devices with channel thicknesses of 100, 50, and 8 nm, respectively. Typical *n*-type conduction characteristics are confirmed, with a channel formed according to the positive gate voltage, and linear and saturation regions are confirmed.

**Figure 4:**
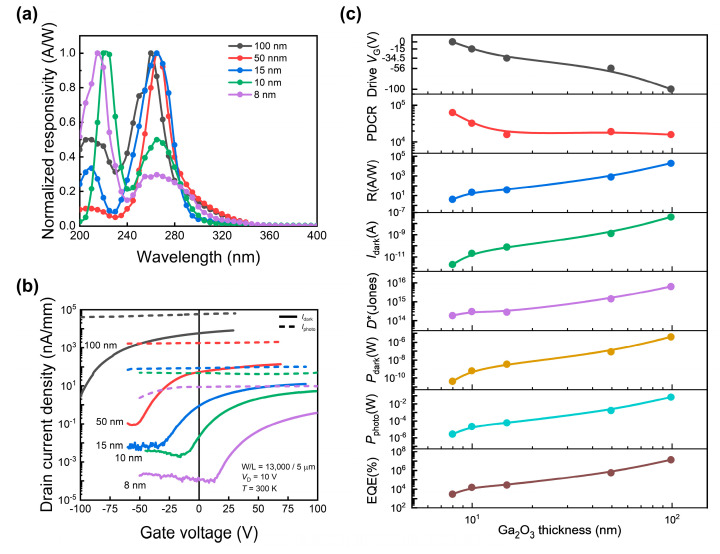
(**a**) Normalized spectral responsivity, and (**b**) drain current density for dark current (*I*_dark_) and photocurrent (*I*_photo_) in transfer curves of fabricated Sn-doped *β*-Ga_2_O_3_ phototransistors according to channel thickness. In (**b**), a DUV monochromatic light source with a wavelength of 210 nm was used, with an optical power density (*P*_in_) of 0.25 μW/mm^2^. (**c**) Photodetector performance comparison results of Sn-doped *β*-Ga_2_O_3_ phototransistors according to Ga_2_O_3_ thickness.

We measured and characterized the optical performance of the Sn-doped *β*-Ga_2_O_3_ phototransistors. Figure 4a shows their normalized spectral responsivity measurements for different channel thicknesses. The 100, 50, and 15 nm channels showed peak photoresponsivity at 260, 265, and 265 nm. However, for the 15, 10, and 8 nm channels, the peak response wavelengths were 220 and 215 nm, respectively. This shift can be attributed to the quantum confinement effect, which increases the bandgap as the film becomes thinner. Additionally, the reduced thickness results in an insufficient absorption depth at longer wavelengths, leading to improved wavelength-selective responsivity. High sensitivity is maintained at shorter wavelengths and sensitivity is suppressed at longer wavelengths. This is advantageous for solar-blind UV detectors. Figure 4b presents the *I*_dark_ and *I*_photo_ transfer curves of Sn-doped *β*-Ga_2_O_3_ phototransistors for each channel thickness, measured under 210 nm monochromatic light, to compare DUV response characteristics. Photocurrent measurements were performed after more than 500 s of light irradiation to ensure saturation. The results demonstrate excellent light-response characteristics in the DUV region for all thicknesses. Figure 4c compares the photodetector performance parameters for each Ga_2_O_3_ thickness, derived from the *I*_dark_ and *I*_photo_ results shown in Figure 4b. The drive *V*_G_ was selected as the smallest *V*_G_ below the threshold voltage. *P*_dark_ and *P*_photo_ represent the device power consumption under dark and illuminated conditions and were calculated as $P=I_{D}\cdot V_{D}+I_{G}\cdot \;\frac{{\left|V_{G}\right|}^{2}+{\left|V_{G}-V_{D}\right|}^{2}}{\left|V_{G}\right|+\left|V_{G}-V_{D}\right|}$, where $I_{D}$ and $V_{D}$ are drain current and voltage, and $I_{G}$ and $V_{G}$ are gate current and voltage, respectively [52,53]. As the Ga_2_O_3_ channel thickness decreased, the driving gate voltage approached zero, and the dark current decreased. For devices with a Ga_2_O_3_ thickness of 8 nm, *I*_dark_ had the smallest value of 1.84 pA. This performance improvement was confirmed by the increased photo-to-dark current ratio (PDCR) caused by the reduced dark current. Conversely, with thicker channels, parameters such as responsivity (*R*), detectivity (D*), and external quantum efficiency (EQE), which are primarily related to the absolute value of the photocurrent, exhibited superior performance. Table 4 presents the detailed values of performance parameters of Sn-doped *β*-Ga_2_O_3_ phototransistors for each thickness tested in light and dark environments. Mobility was calculated using the $\mu =\frac{L}{W}\cdot \frac{1}{C_{ox}}\cdot \frac{\partial I_{D}}{\partial V_{G}}\cdot \frac{1}{V_{D}}$ formula under dark conditions. Here, the relative permittivity of the SiO_2_ was calculated to be 3.9. The on–off ratio was calculated as the ratio of the maximum drain current to the minimum drain current in the transfer curves under dark conditions. For the 100 nm device, no clear device turn-off was observed within the measurement range, so the on–off ratio was not defined. In conclusion, the thickness of the Ga_2_O_3_ light-receiving channel is directly related to the photodetector performance parameters. These findings provide valuable insights and guidance for selecting the optimal thickness for the intended use of DUV phototransistors.

**Figure 5:**
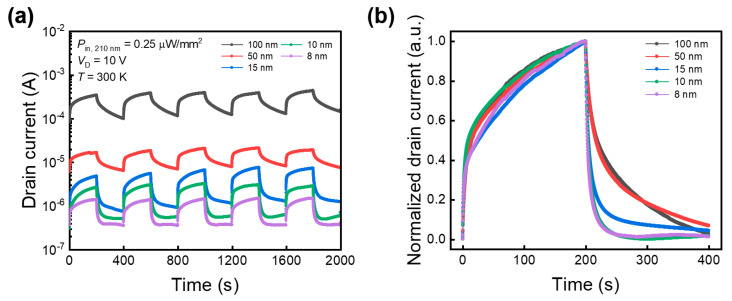
(**a**) Time-dependent on–off response characteristics of the Sn-doped *β*-Ga_2_O_3_ phototransistors device by thickness measured by applying a 210 nm monochromatic light source. One cycle was performed over 400 s. (**b**) Normalized drain current comparison results for one cycle of optical switching investigation for each Ga_2_O_3_ thickness.

Figure 5a shows the time-dependent on–off response characteristics of the Sn-doped *β*-Ga_2_O_3_ phototransistors with varying thicknesses, measured under a 210 nm monochromatic light source. Owing to the polycrystalline structure, complete saturation takes hundreds of seconds because of scattering events and defects. However, the devices exhibited stable operational characteristics even after repeated cycling. Figure 5b normalizes one cycle to compare the light response speeds for each thickness. Although there was no significant difference in the rate of increase for different thicknesses, the decay rate increased as the thickness decreased. In bulk materials, charge carriers are scattered from various sources, including lattice vibrations (phonons), impurities, and defects [54]. However, when the light-absorption channel becomes sufficiently thin to constrain the dimensions, the carriers are confined to a single plane, reducing the number of scattering events. This reduced dimensionality suppresses electron–phonon interactions, leading to higher decay rates [55,56]. Therefore, decreasing the thickness of the Ga_2_O_3_ channel is advantageous in terms of light response speed. An improvement in response speed was confirmed as the thickness decreased, but from the perspective of commercialization of the device, a faster response speed is required. Accordingly, follow-up research is needed to improve the crystallinity of the thin films and suppress defects that affect photocurrent delay. From another perspective, the saturation and recovery delay of the photocurrent have potential applications in artificial neural networks where linearity and symmetry of the signal are significant, such as image generation and neural networks [57,58]. Table 5 shows the performance parameters and drive *V*_G_ of the Sn-doped *β*-Ga_2_O_3_ phototransistors with 8 nm and 100 nm thicknesses, compared with other gallium oxide-based devices. The proposed Ga_2_O_3_ device has a usable performance level compared to the conventional results. When the channel thickness of the proposed device is reduced to 8 nm, the drive gate voltage can be set to zero due to the transition from d-mode to e-mode operation. As confirmed from the previously published results, the Ga_2_O_3_ channel thickness correlates with the drive *V*_G_. These results are significant in that the operation mode switching characteristics of Ga_2_O_3_ transistors, engineered from the perspective of optical devices, have practical implications in the field of semiconductor devices and optical engineering.

## 4. Conclusions

A Sn-doped polycrystalline *β*-Ga_2_O_3_-based phototransistor was fabricated through a sputtering process. The device was optimized from the d-mode to the e-mode through *V*_T_ modulation achieved by Sn doping and channel thickness tuning. Additionally, the diffusion of Ga generated during the process was considered to affect the *V*_T_ shift. Through TCAD device simulation, the transfer curves for each thickness in the dark were successfully matched, and the operation mode mechanism was analyzed. The proposed *β*-Ga_2_O_3_-based phototransistor was proven suitable for DUV detection through optical responsivity measurements. Moreover, as the Ga_2_O_3_ channel thickness decreased, the power consumption and response speed improved. However, the *R*, D*, and EQE performance, which are related to the absolute value of the photocurrent, improved with thickness. This study provides valuable insights and guidance for selecting the optimal thickness for the intended applications of DUV phototransistors. With the increasing potential of Ga_2_O_3_ in the power semiconductor field, the importance of achieving e-mode operation has also become a focal point. However, the quantitative and detailed mechanisms underlying e-mode and d-mode operation in the Ga_2_O_3_ field remain unresolved challenges. The *V*_T_ control and optimization proposed in this study, along with the corresponding simulation analysis, will contribute to the technological maturation of the Ga_2_O_3_ field. Recently, there have been efforts to utilize machine learning algorithms to determine the optimal conditions for the devices. However, foundational datasets, like those proposed in this study, are essential for effective optimization using machine learning algorithms [69,70]. We anticipate that this research will serve as valuable data when machine learning is applied to various Ga_2_O_3_ applications in the future. Furthermore, as machine learning results accumulate, the key parameters used in device simulations are expected to become sufficiently reliable.

## Figures and Tables

**Table 1:** Comparison of bulk lattice parameters for different major planes in 8 nm- and 100 nm-thick Sn-doped polycrystalline *β*-Ga_2_O_3_.

Planes	Lattice Spacing [Å]		Planes	Lattice Spacing [Å]	
	JCPDS	8 nm Sn-Doped Polycrystalline *β*-Ga_2_O_3_		JCPDS	100 nm Sn-Doped Polycrystalline *β*-Ga_2_O_3_
**(−401)**	2.929	2.920	**(111)**	2.549	2.549
**(−111)**	2.675	2.665	**(−311)**	2.343	2.356
**(002)**	2.817	2.857	**(400)**	2.971	2.998
**(112)**	1.978	1.962	**(401)**	2.403	2.385

**Table 2:** Sn-doped polycrystalline *β*-Ga_2_O_3_ thickness and SOG solution concentration conditions.

*β*-Ga_2_O_3_ Thickness [nm]	SOG Solution Concentration [mM]	Doping Weight
100	25	1
50	21.875	0.875
15	18.75	0.75
10	18.75	0.75
8	12.5	0.5

**Table 3:** Materials physical model parameters used in TCAD simulation of transfer curves of Sn-doped polycrystalline *β*-Ga_2_O_3_ phototransistors.

**Material Parameters**	**Values**	**Unit**	**Source**
Dielectric constant	10	unitless	[45]
Electron affinity	4	eV	[46]
Electron density of state (300 K)	3.72 × 10^18^	cm^−3^	[47]
Electron density of state (300 K)	3.72 × 10^18^	cm^−3^	[47]
**Shockley–Read–Hall Recombination (SRG) Model**	**Values**	**Unit**	**Source**
Specifies SRH lifetime for electrons	1.2 × 10^−8^	s	[48]
Specifies SRH lifetime for holes	1.2 × 10^−8^	s	[48]

**Table 4:** Comparison of performance parameters of each Sn-doped *β*-Ga_2_O_3_ phototransistor with various Ga_2_O_3_ thicknesses.

Parameter	*β*-Ga_2_O_3_ Thickness [nm]				
	100	50	15	10	8
***V*_T_ [V]**	−65.98	−31.67	−1.2	20.8	53.2
**Mobility [cm^2^/V∙s]**	3.81 × 10^−3^	5.23 × 10^−5^	7.6 × 10^−6^	2.89 × 10^−6^	3.94 × 10^−7^
**On–off ratio**	-	1.66 × 10^3^	2.93 × 10^3^	3.7 × 10^3^	4.73 × 10^3^
**Drive *V*_G_ [V]**	−100	−56	−34.5	−15	0
**PDCR**	1.57 × 10^4^	1.90 × 10^4^	1.55 × 10^4^	3.20 × 10^4^	6.19 × 10^4^
***R* [A/W]**	1.73 × 10^4^	7.08 × 10^2^	34.2	19.4	3.7
***I*_dark_ [A]**	3.39 × 10^−8^	1.15 × 10^−9^	6.75 × 10^−11^	1.86 × 10^−11^	1.84 × 10^−12^
D* [Jones]	5.87 × 10^15^	1.31 × 10^15^	2.60 × 10^14^	2.80 × 10^14^	1.71 × 10^14^
***P*_dark_ [W]**	3.20 × 10^−6^	6.78 × 10^−8^	2.84 × 10^−9^	5.03 × 10^−10^	3.31 × 10^−11^
***P*_photo_ [W]**	5.01 × 10^−2^	1.29 × 10^−3^	4.42 × 10^−5^	1.61 × 10^−5^	2.05 × 10^−6^
**EQE [%]**	1.02 × 10^7^	4.18 × 10^5^	2.02 × 10^4^	1.14 × 10^4^	2.19 × 10^3^

**Table 5:** Comparison of performance parameters of Sn-doped *β*-Ga_2_O_3_ phototransistor with reported values from other gallium-oxide-based devices.

Channel Thickness [nm]	Drive *V*_G_ [V]	*R* [A/W]	*I*_dark_ [A]	PDCR	D* [Jones]	Ref.
**8**	0	3.7	1.84 × 10^−12^	6.19 × 10^4^	1.71 × 10^14^	This work
**100**	−100	1.73 × 10^4^	3.39 × 10^−8^	1.57 × 10^4^	5.87 × 10^15^	This work
**280**	−8	2.6 × 10^3^	1.2 × 10^−13^	6 × 10^8^	9.7 × 10^13^	[22]
**270**	−20	2.4 × 10^7^	6.7 × 10^−12^	-	1.7 × 10^15^	[59]
**208**	−30	4.1 × 10^3^	2 × 10^−11^	-	2.57 × 10^13^	[60]
**200**	−5	3 × 10^3^	7 × 10^−13^	1.1 × 10^6^	1.3 × 10^16^	[61]
**267**	−10	2.3 × 10^3^	≈1.5 × 10^−9^	6.67 × 10^3^	1.87 × 10^14^	[62]
**112**	−27	1.43 × 10^7^	≈4.8 × 10^−13^	6.9 × 10^7^	1.1 × 10^19^	[63]
**217**	−10	1.17 × 10^5^	2.7 × 10^−14^	1.08 × 10^7^	1.19 × 10^18^	[64]
**381**	−39.5	1.93 × 10^6^	4.35 × 10^−11^	3.1 × 10^8^	1.9 × 10^15^	[65]
**400**	10	5.67 × 10^3^	≈1 × 10^−12^	5.7 × 10^7^	1.87 × 10^15^	[66]
**59**	0	1 × 10^2^	1 × 10^−13^	≈3 × 10^8^	1 × 10^15^	[67]
**30**	0.2	2.17	1.61 × 10^−12^	≈1 × 10^5^	1.71 × 10^12^	[52]
**142**	−20	4.79 × 10^5^	9.91 × 10^−12^	1 × 10^5^	6.69 × 10^14^	[68]

## Data Availability

The data presented in this study are available on request from the corresponding author.
